# Assessment of the Diagnostic Performance of Fully Automated Hepatitis E Virus (HEV) Antibody Tests

**DOI:** 10.3390/diagnostics14060602

**Published:** 2024-03-12

**Authors:** Anna Eichhorn, Franziska Neumann, Carina Bäumler, Imke Gutsmann, Olaf Grobe, Frieda Schlüter, Sina Müller, Andi Krumbholz

**Affiliations:** 1 DiaSorin Deutschland GmbH, Von-Hevesy-Straße 3, D-63128 Dietzenbach, Germany; 2 Labor Dr. Krause und Kollegen MVZ GmbH, Steenbeker Weg 23, D-24106 Kiel, Germany; 3 Institut für Infektionsmedizin, Christian-Albrechts-Universität zu Kiel und Universitätsklinikum Schleswig-Holstein, Campus Kiel, Brunswiker Straße 4, D-24105 Kiel, Germany

**Keywords:** hepatitis E virus, immunoassays, percentage agreement, polyclonal stimulation

## Abstract

The detection of anti-hepatitis E virus (HEV) antibodies contributes to the diagnosis of hepatitis E. The diagnostic suitability of two automated chemiluminescence immunoassays (CLIAs, LIAISON^®^ MUREX Anti-HEV IgG/Anti-HEV IgM test, DiaSorin) was assessed by comparison with the results of a combination of enzyme immunoassays and immunoblots (*recom*Well HEV IgG/IgM ELISA, *recom*Line HEV IgG/IgM, MIKROGEN). Samples with a deviating result were analyzed with the WANTAI ELISAs. Compared to the *recom*Well ELISAs, the Anti-HEV IgG CLIA had a percentage overall agreement (POA) of 100% (149/149; 95% CI: 97.5–100%) and the Anti-HEV IgM CLIA had a POA of 83.3% (85/102; 95% CI: 74.9–89.3%); considering the *recom*Line HEV IgM results, the POA was 71.7% (38/53; 95% CI: 58.4–82%). The WANTAI test confirmed 52.9% (9/17) of negative CLIA IgMs; HEV RNA was not detectable. Since acute infection with the Epstein–Barr virus (EBV) or human cytomegalovirus (CMV) may influence the results of other serological assays, HEV antibodies were examined in 17 EBV and 2 CMV patients: One had an isolated and probably unspecific HEV IgM in the CLIA, as HEV RNA was not detectable. Both CLIAs are well suited for HEV diagnostics, but isolated IgM should be confirmed. An acute EBV/CMV infection can influence HEV serodiagnostics.

## 1. Introduction

The hepatitis E virus (HEV) belongs to the species *Orthohepevirus A* within the family *Hepeviridae* and has a single-stranded positive-sense ribonucleic acid (RNA) genome. HEV is prevalent worldwide and is considered one of the main causes of viral acute hepatitis [1]. To date, eight HEV genotypes (gts) have been distinguished, which differ in their host tropism and epidemiology [1]. In Germany and some other regions of Europe and North America, gt 3 in particular is endemic. Domestic and wild pigs represent important animal reservoirs for this genotype [1,2]. The most important source of infection for humans is the consumption of raw or insufficiently cooked meat. Other transmission routes include direct animal contact, consumption of water or agricultural products contaminated with manure, organ transplants, and blood transfusions [3]. Under immunosuppression, infections caused by gt 3 (and rarely gt 4) can progress to chronic infections [1]. In contrast, gt 1 and 2 are limited to humans as hosts and are rarely detected in industrialized countries. Infections of these types are considered travel-related, especially since major outbreaks have been reported in regions with poor sanitary conditions [1]. The number of HEV infections reported annually is steadily increasing in many industrialized countries, mainly due to increased awareness among medical staff and the use of more sensitive diagnostic tests [4,5].

Laboratory diagnostics play a central role in the detection of HEV infections and provide information on the spread of HEV [6,7]. According to the guidelines of the European Association for the Study of the Liver, a combination of specific antibodies and viral genome detection is recommended [8]. While HEV RNA can be detected very early in the acute course of infection, the detection of HEV IgM and IgG antibodies provides information on acute and convalescent infections as well as seroprevalence. In immunocompromised patients, reverse-transcription PCR-based (quantitative) detection of HEV RNA is essential, as antibodies are sometimes not measurable [7,8].

With few exceptions, most of the available tests for the detection of HEV antibodies are performed manually in an enzyme-linked immunoassay (ELISA) format [9]. DiaSorin has recently launched fully automated high-throughput tests for the detection of anti-HEV IgG and IgM antibodies [10]. The aim of this study was to evaluate the performance of the DiaSorin LIAISON^®^ MUREX Anti-HEV IgG and IgM assays in comparison with the established and widely used *recom*Well/*recom*Line HEV IgG and IgM ELISAs/immunoblots from MIKROGEN. To our knowledge, there are no data on this topic yet.

## 2. Materials and Methods

### 2.1. Samples

The study was performed with human sera previously characterized with antibody assays from MIKROGEN, which we defined as the reference for the detection of HEV IgG and IgM (Tables S1–S3). In addition, 17 samples with serological evidence of acute Epstein–Barr virus (EBV) infection and two samples with an antibody constellation suggestive of acute human cytomegalovirus (CMV) infection were included to investigate possible IgM cross-reactivity, which may lead to false-positive HEV IgM results and thus to misdiagnosis of acute infection [11,12,13].

All samples were residual samples and, with the exception of the 19 samples mentioned above, were sent to the laboratory of Dr. Krause und Kollegen MVZ GmbH Kiel for serodiagnosis of HEV infection. The samples came from patients of registered doctors from northern Germany and were mainly sent in 2021. The sera were stored in the refrigerator/freezer for several days/weeks after arrival at the laboratory until all tests were completed. Repeated thawing and freezing cycles were avoided as far as possible. Information on clinical symptoms and liver function was not available, which is a limitation of this study.

### 2.2. HEV Assays

The initial HEV antibody status was determined manually using the *recom*Well HEV IgG or HEV IgM ELISA (MIKROGEN GmbH, Neuried, Germany; negative < 20 U/mL, borderline 20 to 24 U/mL, positive > 24 U/mL) on a BEP2000 system (Siemens Healthineers AG, Erlangen, Germany); these screening assays serve as a reference here. According to MIKROGEN, these indirect sandwich ELISAs are based on recombinant proteins expressed from the second open reading frame (ORF2) of HEV gt 1 and 3 and should cover antibody responses against the capsid of HEV gt 1 to 4. Their performance was recently analyzed in detail [14].

Sera in which HEV IgG or IgM was detected via these ELISAs were immunoblotted (*recom*Line HEV IgG/IgM on a Dynablot Plus system from MIKROGEN). The manufacturer spotted different parts of the recombinant capsid protein of gt 1 and 3 and the protein derived from ORF3 on a nitrocellulose strip. Each blot contained a separate cut-off control and was automatically evaluated with a BLOTrix Reader and *recom*Scan software (Version 3.4.166; BioSciTec GmbH, Frankfurt/Main, Germany). This method is designed for the detection of antibodies against HEV gt 1 to 4. The results obtained with a combination of MIKROGEN ELISA and blotting serve here as a second reference.

The sera were retested with the fully automated chemiluminescence immunoassays (CLIAs) LIAISON^®^ MUREX Anti-HEV IgG (DiaSorin Italia S.p.A., Saluggia, Italy; quantitative; positive ≥ 0.3 IU/mL) and LIAISON^®^ MUREX Anti-HEV IgM (qualitative; index ≥ 1.00), which use the gt 1 and 3 capsid proteins as antigens [10]. Available data from some patients with acute infection by HEV gt 1, 2 and 4 show that these tests can reliably detect the corresponding antibodies [10].

Sera with serological evidence of acute EBV infection, i.e., presence of anti-viral capsid antigen (VCA) IgG/IgM and absence of the anti-Epstein–Barr virus nuclear antigen (EBNA)-1 IgG (Alinity i™ EBV VCA IgG, EBV VCA IgM and EBV EBNA-1 IgG Reagent Kits; Abbott, Wiesbaden, Germany), or acute/reactivated CMV infection, i.e., presence of CMV IgM (Abbott Alinity i™ CMV IgM/IgG Reagent Kit, Abbott, Wiesbaden, Germany) were also analyzed with the HEV antibody assays from MIKROGEN and DiaSorin.

Samples with discrepant results were subjected to WANTAI HEV-IgG and WANTAI HEV-IgM ELISAs (Beijing Wantai Biological Pharmacy Enterprise Co., Ltd., Beijing, China), which are known to have particularly high assay sensitivity and specificity [15,16,17]. These assays have a grey zone from 0.9 to 1.1 (sample absorbance value/cut-off, i.e., absorbance value of negative control + 0.16 for IgG or 0.26 for IgM), above which the assay is considered positive. As far as we know from the literature, the WANTAI assays use an antigen derived from a highly conserved region of ORF2 [14]. It was assumed that identical results obtained with the assays from two of the three manufacturers were correct.

Samples with discrepant results were also tested for the presence of HEV RNA using a RealStar^®^ HEV RT–PCR Kit 2.0 (Altona Diagnostics GmbH, Hamburg, Germany). Depending on the RNA extraction method used, this test has a lower detection limit of 49 to 329 IU/mL plasma [18].

All assays are CE-certified for HEV diagnostics and were performed according to the manufacturer’s instructions.

### 2.3. Calculation of LIAISON^®^ MUREX Anti-HEV Immunoassays Performance

The percentage positive, negative and overall agreement (PPA, PNA, POA) of the DiaSorin tests was determined with the help of a four-field table in comparison with the reference method. These parameters and their 95% confidence intervals (CIs) were calculated using freely available software at https://tools.westgard.com/two-by-two-contingency.shtml (see: https://www.westgard.com/lessons/basic-method-validation/879-qualitative-test-clinical-agreement.html; accessed 13 February 2024).

## 3. Results

### 3.1. HEV IgG

The diagnostic performance of the LIAISON^®^ MUREX Anti-HEV IgG immunoassay was evaluated in comparison with that of the MIKROGEN *recom*Well HEV IgG, which served as the reference assay. For this purpose, 100 sera previously reactive in the *recom*Well HEV IgG ELISA (isolated IgG positive/borderline, *N* = 60; IgG and IgM positive/borderline, *N* = 40) and 49 sera in which no HEV antibodies were detectable in both the *recom*Well HEV IgG and the *recom*Well HEV IgM ELISA were reexamined with the LIAISON^®^ IgG assay. Table 1 shows qualitative agreement between the results of both assays. The PPA was 100% (100/100; 95% CI: 96.3–100%), the PNA was 100% (49/49; 95% CI: 92.7–100%), and the POA was 100% (149/149; 95% CI: 97.5–100%). As the negative HEV IgG results of the *recom*Well HEV IgG test were not checked again with the MIKROGEN *recom*Line immunoblot, these missing data cannot be included in the calculation of the percentage agreement. Consideration of the immunoblot data had no influence on the agreement of the positive test results.

**Figure 1:**
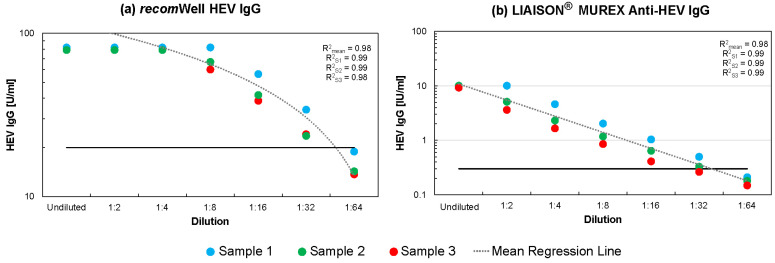
**Linearity of HEV IgG determination across multiple serum dilution levels.** Three samples in which HEV IgG was detectable at high levels were serially diluted in HEV IgG-negative serum and measured in duplicate. Mean HEV IgG concentrations are given. The 1:64 dilution was negative according to both the MIKROGEN *recom*Well HEV IgG assay (<20 U/mL, black horizontal line) (**a**) and the DiaSorin LIAISON^®^ MUREX Anti-HEV IgG assay (<0.3 IU/mL, black horizontal line) (**b**). The 1:16 dilution was consistently found to be positive in the *recom*Line HEV IgG assay, while at the 1:32 dilution, only one of the three serum samples was positive in the immunoblot. The coefficient of determination (R^2^) was calculated both for the regression lines of the three different serum dilution series and for a regression line averaged from these data. When calculating R^2^ in Figure 1a, only the linear range from a dilution level of 1:8 was taken into account. The raw data can be found in Table S4.

The linearity of the HEV IgG measurements was demonstrated for three samples, in which a very high IgG concentration of approximately 80 U/mL was detected via the *recom*Well HEV IgG assay. These samples were serially diluted with HEV IgG-negative serum and measured in duplicate using the *recom*Well IgG and LIAISON^®^ MUREX Anti-HEV IgG assays. After a serum dilution of 1:8, the HEV IgG concentration in the *recom*Well ELISA decreased linearly. In contrast, the LIAISON^®^ MUREX Anti-HEV IgG assay showed a linear decrease in the HEV IgG concentration across all dilution levels tested (Figure 1).

### 3.2. HEV IgM

The diagnostic performance of the DiaSorin LIAISON^®^ MUREX Anti-HEV IgM test was demonstrated using 102 serum samples previously characterized with the MIKROGEN *recom*Well HEV IgM assay. Table 2 shows qualitative agreement between the results of both assays. The PPA was estimated with 67.9% (36/53; 95% CI: 54.5–78.9%), the PNA was 100% (49/49; 95% CI: 92.7–100%) and the POA was 83.3% (85/102; 95% CI: 74.9–89.3%). A deviating result was obtained for 17 samples, which was also confirmed after retesting with both assays. Therefore, the WANTAI HEV-IgM immunoassay was used to evaluate these serum samples. A total of 9 of the 17 samples were also classified as HEV IgM negative by the WANTAI assay. HEV RNA was not detected in any of the 17 discrepant serum samples by RT–PCR (Table 3). When immunoblot results were considered (Table 2), the PPA was 88.9% (24/27; 95% CI: 71.9–96.1%), the PNA was 53.8% (14/26; 95% CI: 35.5–71.2%) and the POA was 71.7% (38/53; 95% CI: 58.4–82%).

### 3.3. Presence of (Non-Specific) HEV Antibodies in Patients with Acute EBV or CMV Infection

Seventeen samples with serological signs of acute EBV infection and two samples with evidence of CMV infection were retested for HEV IgG/IgM. The same three EBV blood samples (i.e., Nos. 1, 7, 8) were identified as HEV IgG-positive in the assays of the three manufacturers. Among them, sample No. 1 was also positive for HEV IgM in their ELISAs/CLIA. In addition, two different acute EBV serum samples, Nos. 8 and 2, were identified as HEV IgM carriers using the MIKROGEN ELISA (No. 8) or the DiaSorin CLIA (No. 2). However, no HEV IgG was detectable in the latter sample (isolated HEV IgM). For the two CMV serum samples, the MIKROGEN and DiaSorin tests gave identical results. HEV RNA was not detected in any of the EBV/CMV samples that tested positive for HEV antibodies (Table 4).

## 4. Discussion

Immunoassays for the detection of HEV IgM and IgG antibodies are widely used due to their ease of use and comparatively low cost. The problem, however, is that the tests have different sensitivities and specificities and give qualitative, semiquantitative, or quantitative results [7,9,12,16,17,19,20]. The differential performance of HEV IgG assays has important implications for seroprevalence estimates [21]. A World Health Organization (WHO) reference serum (NISBSC 95/584) for standardizing HEV antibody tests has been available for several years [22] and could help to improve assay comparability.

In the present study, HEV IgG and IgM tests from two well-known manufacturers were directly compared and the percentage of positive, negative, and overall agreement of the qualitative test results was calculated. If individual results differed, antibody tests from a third, reputable manufacturer were used to make a decision. All assays are approved for routine diagnostics and there are extensive data from the manufacturers and from various studies on their diagnostic quality, although not in a direct comparison of MIKROGEN and DiaSorin. We considered the ELISAs and immunoblots from MIKROGEN, which have been used in diagnostics for some time, as reference tests. 

The DiaSorin LIAISON^®^ MUREX Anti-HEV IgG CLIA test showed qualitative results consistent with those of the MIKROGEN *recom*Well HEV IgG ELISA, which was used as a reference (Table 1). These assays also provide quantitative results. The LIAISON^®^ MUREX Anti-HEV IgG test is aligned with the WHO standard. In general, both assays appear to be suitable for seroprevalence studies. The CLIA has the advantage of being fully automated. This assay was recently used in a Brazilian HEV seroprevalence study [23].

Good linearity was previously reported for the MIKROGEN IgG test [17]. The HEV IgG assay from DiaSorin has a linear range that goes beyond this range (Figure 1). The HEV IgG concentration of samples in which >10 IU/mL HEV IgG was determined during the initial measurement can be reliably determined after sufficient dilution. This could be of interest for the assessment of HEV IgG kinetics in the context of scientific studies.

The HEV IgM antibody test results (Table 2) are more heterogeneous and require detailed discussion. The highest number of HEV IgM-positive samples was found with the MIKROGEN *recom*Well HEV ELISA. The comparatively high reactivity of the *recom*Well HEV IgM assay was demonstrated in an earlier study [17]. However, HEV RNA could not be detected in any of the 17 samples that were reactive in this test but not in the DiaSorin assay. In contrast to viral RNA, HEV IgM may not be detectable in the very early stages of infection, while IgM seroconversion may accompany the onset of symptoms and persist for many months thereafter [7,24]. The results of a current study with asymptomatic blood donors demonstrate that only one in four to five viremic donors already have HEV IgM antibodies at the time of the first RNA detection [25]. Therefore, at least in these 17 non-viremic patients, post-acute infection status, persistent IgM or even false-positive IgM detection are assumed. When the *recom*Well HEV IgM ELISA was used in combination with the HEV IgM *recom*Line immunoblot, as recommended by MIKROGEN, the number of IgM detections decreased by approximately 50% (27 out of 53 positive samples were confirmed by immunoblotting). A total of 8 of the 17 samples found to be reactive by the *recom*Well HEV IgM ELISA were concordantly negative by the *recom*Line HEV IgM blot and by the DiaSorin and WANTAI IgM assays (Table 3). Recently, good agreement was reported between HEV antibody tests from the latter two manufacturers [10]. The indices of samples No. 13 and No. 21 were close to 1.00, above which the DiaSorin CLIA is categorized as IgM positive (Table 3). The data in Table 2 and Table 3 show that negative results of the DiaSorin LIAISON^®^ MUREX Anti-HEV IgM test do not generally have to be confirmed. Only samples with indices close to 1.00 could provide a reason for confirmatory/follow-up diagnostics. In 4 of the 53 IgM-positive serum samples, no corresponding IgG antibodies were detectable (quoted as isolated HEV IgM). Two of these samples, No. 20 and No. 40, were reactive in the *recom*Well HEV IgM ELISA, the *recom*Line HEV IgM immunoblot and the CLIA, while two samples, No. 26 and No. 28, were reactive only in the *recom*Well HEV IgM test (Table S2 and Table 3). The latter two samples were suspected to be false positives for HEV IgM. However, we do not have information on the clinical picture on which HEV serology was requested. These findings underscore the importance of the *recom*Line HEV IgM immunoblot for the verification of reactive MIKROGEN HEV IgM ELISA results. In particular, the detection of isolated HEV IgM (without concomitant HEV IgG) should lead to confirmatory and follow-up tests [8,11]. The exclusion of viremia by the application of nucleic acid amplification techniques (NAAT) like PCR may be very useful in these cases [7]. 

The investigation of a limited number of samples with serologically suspected acute EBV/CMV infection revealed a possible false-reactive HEV IgM (Sample No. 2, Table 4), confirming the results of previous studies [12,13,26]. For the MIKROGEN *recom*Line HEV IgM assay, for example, Dichtl et al. reported isolated HEV IgM reactivity in 2 of 12 patients with acute EBV infection [12]. This phenomenon is most likely due to polyclonal B-cell stimulation associated with herpesvirus infection [26]. Therefore, in addition to HEV IgM confirmatory and follow-up testing (including NAAT), other infections should be excluded as appropriate [11].

The significance of this study is limited by the lack of information on the clinical symptoms that led to the request for an HEV antibody test. Furthermore, the number of samples included is comparatively small, but in the same order of magnitude as in several other studies on the performance of various HEV antibody tests [7]. Not all samples were analyzed in the HEV PCR. However, at least all that were reactive in just one HEV IgM assay were tested free of HEV RNA, so that a very early HEV infection status is unlikely. We therefore consider the comparison with antibody tests, which have long been used in routine serological diagnostics and have been characterized in a number of studies, to be suitable for an indicative evaluation of the diagnostic performance of the new CLIAs, although examination of a larger number of samples would be desirable.

## 5. Conclusions

The fully automated DiaSorin LIAISON^®^ MUREX Anti-HEV IgG and IgM assays are sensitive and specific high-throughput tests with good performance. Both tests are useful for the diagnosis of acute and convalescent HEV infections in immunocompetent patients. The HEV IgG CLIA is also suitable for seroprevalence studies. The detection of HEV IgM does not necessarily mean that an acute HEV infection is present. In particular, an isolated HEV IgM should be confirmed by follow-up and alternative tests including NAAT. HEV IgM test results may be biased in patients with acute EBV/CMV infection.

## Figures and Tables

**Table 1:** **Qualitative agreement of the MIKROGEN *recom*Well/*recom*Line HEV IgG and the DiaSorin LIAISON^®^ MUREX Anti-HEV IgG immunoassays.** The raw data can be found in Tables S1 and S3.

		MIKROGEN *recom*Well HEV IgG		
		Positive/Borderline (Positive >24 U/mL Borderline 20 U/mL to 24 U/mL)	Negative	Total
DiaSorin LIAISON^®^ MUREX Anti-HEV IgG	Positive (≥0.3 IU/mL)	97/3	0	100
	Negative	0	49	49
	Total	100	49	149
		MIKROGEN *recom*Well HEV IgG & *recom*Line HEV IgG		
		Positive	Negative	Total
DiaSorin LIAISON^®^ MUREX Anti-HEV IgG	Positive (≥0.3 IU/mL)	100	-	100
	Negative	0	-	0
	Total	100	-	100

**Table 2:** **Qualitative agreement of the MIKROGEN *recom*Well/*recom*Line HEV IgM and the DiaSorin LIAISON^®^ MUREX Anti-HEV IgM immunoassays.** The raw data can be found in Tables S2 and S3.

		MIKROGEN *recom*Well HEV IgM		
		Positive/Borderline (Positive >24 U/mL Borderline 20 U/mL to 24 U/mL)	Negative	Total
DiaSorin LIAISON^®^ MUREX Anti-HEV IgM	Positive (Index ≥ 1.00)	35/1	0	36
	Negative	13/4 *	49	66
	Total	53	49	102
		MIKROGEN *recom*Well HEV IgM & *recom*Line HEV IgM		
		Positive	Negative	Total
DiaSorin LIAISON^®^ MUREX Anti-HEV IgM	Positive (Index ≥ 1.00)	24	12	36
	Negative	3	14	17
	Total	27	26	53

* HEV RNA was not detected in these 17 samples by RT-PCR (see Table 3).

**Table 3:** **Characterization of 17 sera with a different result in the DiaSorin LIAISON^®^ MUREX Anti-HEV IgM assay compared to the MIKROGEN *recom*Well HEV IgM test used as a reference.** These samples were tested in duplicate with both assays (mean values are given for each test) and re-evaluated with the WANTAI HEV-IgM assay. The samples are sorted according to the sample/cut-off values of the WANTAI HEV-IgM ELISA (data from Table S2). Abbreviations: No., number; qual., qualitative; +, positive; (+), borderline; −, negative.

	MIKROGEN *recom*Well HEV IgM		MIKROGEN *recom*Line HEV IgM	DiaSorin LIAISON^®^ MUREX Anti-HEV IgM		WANTAI HEV-IgM ELISA		RealStar^®^ HEV RT-PCR Kit 2.0	MIKROGEN *recom*Well HEV IgG	
	Positive (>24 U/mL) Borderline (20 U/mL to 24 U/mL)			Positive (Index ≥ 1.00)		Positive (Sample/Cut-Off ≥ 1.1)			Positive (>24 U/mL)	
No.	[U/mL]	Qual.	Qual.	[Index]	Qual.	S/CO	Qual.	HEV RNA	[U/mL]	Qual.
8	43.0	+	−	0.5	−	4.0	+	−	98.8	+
13	25.0	+	+	0.9	−	2.6	+	−	>125	+
25	22.1	(+)	−	0.7	−	1.7	+	−	107.0	+
39	34.6	+	−	0.8	−	1.7	+	−	>125	+
9	25.9	+	−	0.7	−	1.2	+	−	103.2	+
44	31.6	+	−	0.6	−	1.2	+	−	101.7	+
51	33.0	+	−	0.8	−	1.1	+	−	106.7	+
21	28.6	+	+	0.9	−	1.1	+	−	96.0	+
48	26.0	+	−	0.7	−	0.7	−	−	85.5	+
6	37.2	+	−	0.8	−	0.4	−	−	98.8	+
5	37.9	+	−	0.5	−	0.3	−	−	98.8	+
30	23.9	(+)	+	0.6	−	0.2	−	−	32.1	+
32	22.4	(+)	−	0.5	−	0.1	−	−	92.4	+
26	27.4	+	−	<0.1	−	<0.1	−	−	4.0	−
28	24.5	+	−	<0.1	−	<0.1	−	−	2.3	−
43	24.7	+	−	0.8	−	<0.1	−	−	63.9	+
49	23.8	(+)	−	0.6	−	<0.1	−	−	49.1	+

**Table 4:** **Influence of an acute Epstein–Barr virus (EBV) or human cytomegalovirus (CMV) infection on the results of HEV antibody tests.** Samples with serological evidence for an acute EBV (N = 17) or CMV (N = 2) infection were tested for the presence of HEV antibodies. In sample No. 2, isolated IgM (without HEV IgG) was detected with only one test, which is why the result was evaluated as most likely false reactive. The measured raw values are given in brackets. Abbreviations: Infect., infection; No., number; +, positive; (+), borderline; −, negative; n.t., not tested; qual., qualitative.

		HEV IgG				HEV IgM				HEV RNA
		MIKROGEN *recom*Well	MIKROGEN *recom*Line	DiaSorin LIAISON^®^ MUREX	WANTAI	MIKROGEN *recom*Well	MIKROGEN *recom*Line	DiaSorin LIAISON^®^ MUREX	WANTAI	RealStar^®^ HEV RT-PCR Kit 2.0
		Positive (>24 U/mL)		Positive (≥0.3 IU/mL)	Positive (Sample/ Cut-Off ≥ 1.1)	Positive (>24 U/mL) Borderline (20 U/mL to 24 U/mL)		Positive (Index ≥ 1.00)	Positive (Sample/ Cut-Off ≥ 1.1)	
No.	Infect.	Qual. (U/mL)	Qual.	Qual. (IU/mL)	Qual. (S/CO)	Qual. (U/mL)	Qual.	Qual. (Index)	Qual. (S/CO)	RNA
1	EBV	+(>125)	+	+(>10)	+(15.8)	+(31.6)	−	+(1.1)	+(5.5)	−
2	EBV	−(3.0)	n.t.	−(<0.1)	−(<0.1)	−(9.0)	n.t.	+(1.6)	−(0.1)	−
3	EBV	−(2.0)	n.t.	−(<0.1)	−(<0.1)	−(4.5)	n.t.	−(0.2)	−(0.2)	n.t.
4	EBV	−(3.9)	n.t.	−(<0.1)	−(<0.1)	−(1.9)	n.t.	−(<0.1)	−(<0.1)	n.t.
5	EBV	−(3.1)	n.t.	−(<0.1)	−(<0.1)	−(3.0)	n.t.	−(0.2)	−(<0.1)	n.t.
6	EBV	−(2.1)	n.t.	−(<0.1)	−(<0.1)	−(3.0)	n.t.	−(0.1)	−(0.1)	n.t.
7	EBV	+(50.3)	+	+(1.1)	+(12.8)	−(13.4)	n.t.	−(0.5)	−(0.6)	−
8	EBV	+(44.8)	+	+(1.4)	+(10.8)	(+)(20.4)	−	−(0.6)	−(<0.1)	−
9	EBV	−(3.7)	n.t.	−(<0.1)	−(<0.1)	−(2.6)	n.t.	−(0.2)	−(<0.1)	n.t.
10	EBV	−(3.0)	n.t.	−(<0.1)	−(<0.1)	−(7.3)	n.t.	−(0.2)	−(<0.1)	n.t.
11	EBV	−(5.4)	n.t.	−(<0.1)	−(<0.1)	−(4.5)	n.t.	−(0.9)	−(<0.1)	n.t.
12	EBV	−(3.6)	n.t.	−(<0.1)	−(<0.1)	−(6.7)	n.t.	−(0.2)	−(<0.1)	n.t.
13	EBV	−(3.0)	n.t.	−(<0.1)	−(<0.1)	−(5.1)	n.t.	−(0.6)	−(0.1)	n.t.
14	EBV	−(4.0)	n.t.	−(<0.1)	−(<0.1)	−(2.1)	n.t.	−(0.1)	−(0.1)	n.t.
15	EBV	−(2.7)	n.t.	−(<0.1)	−(<0.1)	−(2.7)	n.t.	−(0.7)	−(<0.1)	n.t.
16	EBV	−(5.1)	n.t.	−(<0.1)	−(<0.1)	−(7.7)	n.t.	−(0.1)	−(<0.1)	n.t.
17	EBV	−(3.4)	n.t.	−(<0.1)	−(<0.1)	−(6.7)	n.t.	−(<0.1)	−(<0.1)	n.t.
18	CMV	+(39.5)	n.t.	+(0.5)	n.t.	−(16.9)	n.t.	−(0.5)	n.t.	−
19	CMV	−(6.9)	n.t.	−(<0.1)	−(<0.1)	−(11.2)	n.t.	−(0.2)	−(<0.1)	n.t.

## Data Availability

All the data can be found in the manuscript or in the Supporting Information.

## Supplementary Materials

The following supporting information can be downloaded at: https://www.mdpi.com/article/10.3390/diagnostics14060602/s1, Table S1: Raw data for the comparison of the MIKROGEN (reference) and DiaSorin HEV IgG immunoassays; Table S2: Raw data for the comparison of the MIKROGEN (reference), DiaSorin and WANTAI HEV-IgM immunoassays; Table S3: Raw data for the HEV antibody negative samples included in the study; Table S4: Raw data for the HEV IgG linearity studies.
