# Supplementary material for: Assessment of the Diagnostic Performance of Fully Automated Hepatitis E Virus (HEV) Antibody Tests
Source: Diagnostics (Basel). 2024 Mar 12;14(6):602. doi: 10.3390/diagnostics14060602 (PMC10969403; doi:10.3390/diagnostics14060602)
Supplement: Supplementary file 1 [file diagnostics-14-00602-s001.zip › diagnostics-2906972-supplementary.pdf]

Supplementary materials

Assessment of the Diagnostic Performance of Fully Automated Hepatitis E Virus (HEV) Antibody Tests

Anna Eichhorn[1#], Franziska Neumann[2#], Carina Bäumler[2], Imke Gutsmann[3], Olaf Grobe[2], Frieda Schlüter[2], Sina Müller[2], Andi Krumbholz[2,3*]

1 DiaSorin Deutschland GmbH, Von-Hevesy-Straße 3, D-63128 Dietzenbach, Germany

2 Labor Dr. Krause und Kollegen MVZ GmbH, Steenbeker Weg 23, D-24106 Kiel, Germany

3 Institut für Infektionsmedizin, Christian-Albrechts-Universität zu Kiel und Universitätsklinikum Schleswig Holstein, Campus Kiel, Brunswiker Straße 4, D-24105 Kiel, Germany

[#] both first authors contributed equally

* corresponding author: Andi Krumbholz, MD

krumbholz@infmed.uni-kiel.de

krumbholz@labor-krause.de

ORCID: 0000-0002-1094-350X (Andi Krumbholz)

Table S1: Raw data for the comparison of the MIKROGEN (reference) and DiaSorin HEV IgG immunoassays. Abbreviations: No., number; qual., qualitative; +, positive; (+), borderline.

No.	**MIKROGEN** *recom*Well HEV IgG (Positive > 24 U/ml Borderline 20 U/ml to 24 U/ml)		**MIKROGEN** *recom*Line HEV IgG	**DiaSorin** LIAISON® MUREX Anti-HEV IgG (Positive ≥ 0.3 IU/ml)	
	[U/ml]	qual.	qual.	[IU/ml]	qual.
1	29.0	+	+	0.5	+
2	80.4	+	+	1.3	+
3	79.7	+	+	2.4	+
4	>125	+	+	>10	+
5	>125	+	+	>10	+
6	113.8	+	+	4.5	+
7	>125	+	+	3.7	+
8	>125	+	+	>10	+
9	89.3	+	+	2.4	+
10	103.2	+	+	>10	+
11	103.2	+	+	5.4	+
12	103.2	+	+	>10	+
13	71.4	+	+	1.3	+
14	98.8	+	+	2.9	+
15	91.2	+	+	3.4	+
16	80.0	+	+	1.9	+
17	98.8	+	+	>10	+
18	89.4	+	+	3.1	+
19	35.1	+	+	0.8	+
20	92.3	+	+	>10	+
21	25.4	+	+	0.5	+
22	92.3	+	+	>10	+
23	183.2	+	+	>10	+
24	40.7	+	+	0.6	+
25	59.5	+	+	1.1	+
26	54.3	+	+	0.8	+
27	69.4	+	+	1.1	+
28	107.0	+	+	>10	+
29	107.0	+	+	>10	+
30	96.0	+	+	3.6	+
31	26.1	+	+	0.7	+
32	107.0	+	+	4.7	+
33	107.0	+	+	>10	+
34	117.5	+	+	3.8	+
35	117.5	+	+	>10	+
36	71.7	+	+	1.3	+
37	82.3	+	+	1.5	+
38	32.1	+	+	0.5	+
39	98.4	+	+	6.3	+
40	98.4	+	+	>10	+
41	34.4	+	+	0.49	+
42	40.9	+	+	1.5	+
43	79.4	+	+	1.2	+
44	98.4	+	+	3.5	+
45	46.2	+	+	0.8	+
46	96.4	+	+	4.4	+
47	96.4	+	+	>10	+
48	41.2	+	+	0.7	+
49	41.9	+	+	0.8	+
50	31.0	+	+	0.5	+

No.	**MIKROGEN** *recom*Well HEV IgG (Positive > 24 U/ml Borderline 20 U/ml to 24 U/ml)		**MIKROGEN** *recom*Line HEV IgG	**DiaSorin** LIAISON® MUREX Anti-HEV IgG (Positive ≥ 0.3 IU/ml)	
	[U/ml]	qual.	qual.	[IU/ml]	qual.
51	74.4	+	+	1.6	+
52	92.4	+	+	5.9	+
53	27.2	+	+	0.6	+
54	28.1	+	+	0.5	+
55	51.3	+	+	1.1	+
56	97.6	+	+	>10	+
57	97.6	+	+	>10	+
58	97.6	+	+	>10	+
59	23.9	(+)	+	0.4	+
60	>125	+	+	>10	+
61	>125	+	+	6.5	+
62	23.9	(+)	+	0.3	+
63	44.7	+	+	0.8	+
64	73.0	+	+	1.6	+
65	>125	+	+	>10	+
66	>125	+	+	>10	+
67	101.7	+	+	7.2	+
68	101.7	+	+	3.6	+
69	21.2	(+)	+	0.3	+
70	>125	+	+	>10	+
71	>125	+	+	>10	+
72	>125	+	+	>10	+
73	36.1	+	+	0.9	+
74	60.2	+	+	1.5	+
75	49.1	+	+	0.8	+
76	85.5	+	+	1.9	+
77	106.7	+	+	>10	+
78	34.2	+	+	0.6	+
79	86.4	+	+	2.6	+
80	122.7	+	+	8.5	+
81	34.6	+	+	0.6	+
82	35.7	+	+	0.5	+
83	73.3	+	+	1.6	+
84	25.4	+	+	0.4	+
85	>125	+	+	>10	+
86	>125	+	+	5.0	+
87	>125	+	+	>10	+
88	103.2	+	+	9.8	+
89	98.8	+	+	>10	+
90	92.3	+	+	>10	+
91	43.0	+	+	0.7	+
92	38.5	+	+	0.4	+
93	37.8	+	+	0.4	+
94	>125	+	+	>10	+
95	58.5	+	+	1.3	+
96	>125	+	+	5.2	+
97	63.9	+	+	1.4	+
98	84.7	+	+	2.0	+
99	106.7	+	+	>10	+
100	66.3	+	+	1.4	+

Table S2: Raw data for the comparison of the MIKROGEN (reference), DiaSorin and WANTAI HEV-IgM immunoassays. Samples with a discrepant result between the *recom*Well HEV IgM ELISA and the DiaSorin assay were retested with both assays and confirmed with the WANTAI HEV-IgM assay. Abbreviations: No., number; qual., qualitative; +, positive; (+), borderline; -, negative; n.t., not tested.

No.	**MIKROGEN** *recom*Well HEV IgM (Positive > 24 U/ml Borderline 20 U/ml to 24 U/ml)		**MIKROGEN** *recom*Line HEV IgM	**DiaSorin** LIAISON® MUREX Anti-HEV IgM (Positive Index ≥ 1.00)		**WANTAI** HEV-IgM ELISA (Positive sample/cut-off ≥ 1.1 Borderline Sample/cut-off 0.9 to 1.1)		**RealStar®** HEV RT-PCR Kit 2.0	**MIKROGEN** *recom*Well HEV IgG (Positive > 24 U/ml)	
	[U/ml]	qual.	qual.	[Index]	qual.	sample/cut-off	qual.	HEV RNA	[U/ml]	qual.
1	114.5	+	+	3.1	+	n.t.	n.t.	n.t.	85.2	+
2	83.7	+	-	2.8	+	n.t.	n.t.	n.t.	85.2	+
3	>125	+	-	2.9	+	n.t.	n.t.	n.t.	>125	+
4	100.7	+	+	1.7	+	n.t.	n.t.	n.t.	98.7	+
5	37.9	+	-	0.5	-	0.3	-	-	98.8	+
6	37.2	+	-	0.8	-	0.4	-	-	98.8	+
7	68.7	+	+	3.3	+	n.t.	n.t.	n.t.	91.2	+
8	43.0	+	-	0.5	-	4.0	+	-	98.8	+
9	25.9	+	-	0.7	-	1.2	+	-	103.2	+
10	33.7	+	-	1.3	+	n.t.	n.t.	n.t.	89.3	+
11	45.4	+	-	1.1	+	n.t.	n.t.	n.t.	>125	+
12	>125	+	+	9.1	+	n.t.	n.t.	n.t.	>125	+
13	25.0	+	+	0.9	-	2.6	+	-	>125	+
14	>125	+	+	9.1	+	n.t.	n.t.	n.t.	>125	+
15	39.7	+	+	1.6	+	n.t.	n.t.	n.t.	79.7	+
16	93.4	+	+	5.4	+	n.t.	n.t.	n.t.	92.3	+
17	187.5	+	+	6.9	+	n.t.	n.t.	n.t.	183.2	+
18	98.4	+	+	1.7	+	n.t.	n.t.	n.t.	117.5	+
19	57.5	+	-	1.9	+	n.t.	n.t.	n.t.	117.5	+
20	35.7	+	+	1.5	+	n.t.	n.t.	n.t.	14.7	-
21	28.6	+	+	0.9	-	1.1	+	-	96.0	+
22	>125	+	+	4.0	+	n.t.	n.t.	n.t.	107.0	+
23	72.8	+	-	2.2	+	n.t.	n.t.	n.t.	107.0	+
24	34.2	+	-	1.4	+	n.t.	n.t.	n.t.	107.0	+
25	22.1	(+)	-	0.7	-	1.7	+	-	107.0	+
26	27.4	+	-	<0.1	-	<0.1	-	-	4.0	-
27	83.3	+	+	3.2	+	n.t.	n.t.	n.t.	96.4	+
28	24.5	+	-	<0.1	-	<0.1	-	-	2.3	-
29	120.1	+	+	4.6	+	n.t.	n.t.	n.t.	96.4	+
30	23.9	(+)	+	0.6	-	0.2	-	-	32.1	+
31	116.7	+	+	5.9	+	n.t.	n.t.	n.t.	98.4	+
32	22.4	(+)	-	0.5	-	0.1	-	-	92.4	+

No.	**MIKROGEN** *recom*Well HEV IgM (Positive > 24 U/ml Borderline 20 U/ml to 24 U/ml)		**MIKROGEN** *recom*Line HEV IgM	**DiaSorin** LIAISON® MUREX Anti-HEV IgM (Positive Index ≥ 1.00)		**WANTAI** HEV-IgM ELISA (Positive sample/cut-off ≥ 1.1 Borderline Sample/cut-off 0.9 to 1.1)		**RealStar®** HEV RT-PCR Kit 2.0	**MIKROGEN** *recom*Well HEV IgG (Positive > 24 U/ml)	
	[U/ml]	qual.	qual.	[Index]	qual.	sample/cut-off	qual.	HEV RNA	[U/ml]	qual.
33	27.3	+	-	1.8	+	n.t.	n.t.	n.t.	51.3	+
34	104.4	+	+	1.6	+	n.t.	n.t.	n.t.	92.4	+
35	33.3	+	+	1.4	+	n.t.	n.t.	n.t.	51.3	+
36	46.3	+	-	1.1	+	n.t.	n.t.	n.t.	97.6	+
37	43.2	+	-	1.0	+	n.t.	n.t.	n.t.	>125	+
38	>125	+	+	8.7	+	n.t.	n.t.	n.t.	27.5	+
39	34.6	+	-	0.8	-	1.7	+	-	>125	+
40	22.3	(+)	+	1.8	+	n.t.	n.t.	n.t.	3.0	-
41	>125	+	+	6.6	+	n.t.	n.t.	n.t.	>125	+
42	>125	+	+	3.0	+	n.t.	n.t.	n.t.	>125	+
43	24.7	+	-	0.8	-	<0.1	-	-	63.9	+
44	31.6	+	-	0.6	-	1.2	+	-	101.7	+
45	52.0	+	-	1.1	+	n.t.	n.t.	n.t.	101.7	+
46	>125	+	+	7.0	+	n.t.	n.t.	n.t.	>125	+
47	43.0	+	-	1.4	+	n.t.	n.t.	n.t.	>125	+
48	26.0	+	-	0.7	-	0.7	-	-	85.5	+
49	23.8	(+)	-	0.6	-	<0.1	-	-	49.1	+
50	66.1	+	+	2.2	+	n.t.	n.t.	n.t.	106.7	+
51	33.0	+	-	0.8	-	1.1	+	-	106.7	+
52	>125	+	+	8.2	+	n.t.	n.t.	n.t.	122.7	+
53	>125	+	+	2.2	+	n.t.	n.t.	n.t.	>125	+

Table S3: Raw data for the HEV antibody negative samples included in the study.

Number	**MIKROGEN**<br>*recom*Well HEV IgG<br>(Positive<br>> 24 U/ml<br>Borderline<br>20 U/ml to 24 U/ml)	**DiaSorin**<br>LIAISON® MUREX<br>Anti-HEV IgG<br>(Positive ≥ 0.3 IU/ml)	**MIKROGEN**<br>*recom*Well HEV IgM<br>(Positive<br>> 24 U/ml<br>Borderline<br>20 U/ml to 24 U/ml)	**DiaSorin**<br>LIAISON® MUREX<br>Anti-HEV IgM<br>(Positive<br>Index ≥ 1.00)
	[U/ml]	[IU/ml]	[U/ml]	[Index]
1	1.9	<0.1	3.1	<0.1
2	1.5	0.2	2.8	<0.1
3	5.2	<0.1	3.5	<0.1
4	9.2	0.3	2.8	0.2
5	1.5	<0.1	3.8	0.1
6	2.6	<0.1	7.8	<0.1
7	3.4	<0.1	4.3	0.1
8	2.5	<0.1	2.2	<0.1
9	2.1	<0.1	3.0	0.4
10	2.1	<0.1	10.6	<0.1
11	2.7	<0.1	2.2	<0.1
12	2.5	<0.1	5.4	<0.1
13	2.2	<0.1	1.5	<0.1
14	10.3	0.1	5.4	0.2
15	2.8	<0.1	2.0	<0.1
16	3.4	<0.1	1.5	<0.1
17	2.9	<0.1	2.5	<0.1
18	1.7	<0.1	7.9	0.3
19	2.6	<0.1	3.2	<0.1
20	1.9	<0.1	2.5	<0.1
21	8.8	0.1	2.7	<0.1
22	1.8	<0.1	1.9	<0.1
23	1.2	<0.1	1.7	<0.1
24	2.0	<0.1	2.5	<0.1
25	2.2	<0.1	1.8	<0.1
26	1.2	<0.1	2.1	<0.1
27	1.3	<0.1	2.0	<0.1
28	2.2	<0.1	2.4	<0.1
29	1.7	<0.1	2.8	<0.1
30	14.6	0.2	2.1	<0.1
31	11.2	0.1	2.7	<0.1
32	12.0	0.2	2.3	<0.1
33	19.2	0.2	1.8	<0.1
34	2.0	<0.1	2.4	<0.1
35	4.5	<0.1	4.6	<0.1
36	9.9	<0.1	8.2	<0.1
37	5.4	<0.1	4.3	<0.1
38	5.1	0.2	2.6	<0.1
39	6.1	<0.1	4.4	<0.1
40	3.6	<0.1	6.7	0.8
41	2.3	<0.1	7.9	0.4
42	3.6	<0.1	5.8	<0.1
43	5.5	<0.1	4.4	0.1
44	3.4	<0.1	2.3	<0.1
45	4.3	<0.1	5.7	<0.1
46	3.2	<0.1	4.3	0.3
47	3.9	<0.1	2.4	<0.1
48	5.0	0.1	5.2	0.1
49	3.1	<0.1	3.4	<0.1

Table S4: Raw data for the HEV IgG linearity studies. For all three investigated samples individual as well as mean values are given. Some sera dilutions around the cut-off of the *recom*Well HEV IgG test were also investigated with the *recom*Line HEV IgG immunoblot. Abbreviations: +, positive; -, negative; n.t., not tested.

Sample	Dilution	MIKROGEN *recom*Well HEV IgG Positive > 24 U/ml Borderline 20 U/ml to 24 U/ml [U/ml]	MIKROGEN *recom*Well HEV IgG Mean [U/ml]	MIKROGEN *recom*Line HEV IgG	DiaSorin LIAISON® MUREX Anti-HEV IgG Positive ≥ 0.3 IU/ml [IU/ml]	DiaSorin LIAISON® MUREX Anti-HEV IgG Mean [IU/ml]
# 1	undiluted	81.9	81.9	n.t.	>10	>10
		81.9			>10	
	1:2	81.9	81.9	n.t.	9.5	>10
		81.9			>10	
	1:4	81.9	81.9	n.t.	4.6	4.6
		81.9			4.6	
	1:8	81.9	81.9	n.t.	2.0	2.0
		81.9			2.0	
	1:16	57.7	56.4	+	1.0	1.0
		55.0			1.0	
	1:32	34.2	34.1	-	0.5	0.5
		34.1			0.5	
	1:64	19.6	18.9	-	0.2	0.2
		18.2			0.2	
# 2	undiluted	79.0	79.0	n.t.	>10	>10
		79.0			>10	
	1:2	79.0	79.0	n.t.	4.7	5.1
		79.0			5.5	
	1:4	79.0	79.0	n.t.	2.3	2.3
		79.0			2.3	
	1:8	65.9	66.9	n.t.	1.1	1.2
		67.8			1.2	
	1:16	44.2	42.0	+	0.6	0.6
		39.7			0.6	
	1:32	24.6	23.6	+	0.3	0.4
		22.5			0.4	
	1:64	14.0	14.3	n.t.	0.2	0.2
		14.6			0.2	
# 3	undiluted	79.0	79.0	n.t.	9.4	9.2
		79.0			9.1	
	1:2	79.0	79.0	n.t.	3.8	3.6
		79.0			3.4	
	1:4	79.0	79.0	n.t.	1.7	1.7
		79.0			1.6	
	1:8	61.4	60.0	n.t.	0.9	0.9
		58.7			0.8	
	1:16	39.0	38.7	+	0.4	0.4
		38.3			0.4	
	1:32	24.6	24.1	-	0.3	0.3
		23.5			0.3	
	1:64	13.9	13.7	n.t.	0.1	0.2
		13.5			0.2	
